# Urbanization and malaria have a contextual relationship in endemic areas: A temporal and spatial study in Ghana

**DOI:** 10.1371/journal.pgph.0002871

**Published:** 2024-05-30

**Authors:** Merveille Koissi Savi, Bhartendu Pandey, Anshuman Swain, Jeongki Lim, Daniel Callo-Concha, Gbedegnon Roseric Azondekon, Mohammed Wahjib, Christian Borgemeister

**Affiliations:** 1 Center for Development Research (ZEF), University of Bonn, North Rhine-Westphalia, Germany; 2 Department of Medical Oncology, Dana Farber Cancer Institute, Harvard School of Medicine, Boston, Massachusetts, United States of America; 3 Department of Civil & Environmental Engineering, Princeton University, Princeton, New Jersey, United States of America; 4 Oak Ridge National Laboratory, Oak Ridge, Tennessee, United States of America; 5 Department of Biology, University of Maryland, College Park, Maryland, United States of America; 6 Department of Organismic and Evolutionary Biology, Harvard University, Cambridge, Massachusetts, United States of America; 7 Parsons School of Design, The New School, New York, New York, United States of America; 8 University of Koblenz-Landau, Institute for Environmental Sciences, North Rhine-Westphalia, German; 9 Centre de Recherches Entomologiques de Cotonou, Littoral, Benin; 10 National Malaria Control Programme, Ministry of Health, Accra, Ghana; Georgetown University, UNITED STATES

## Abstract

In West Africa, malaria is one of the leading causes of disease-induced deaths. Existing studies indicate that as urbanization increases, there is corresponding decrease in malaria prevalence. However, in malaria-endemic areas, the prevalence in some rural areas is sometimes lower than in some peri-urban and urban areas. Therefore, the relationship between the degree of urbanization, the impact of living in urban areas, and the prevalence of malaria remains unclear. This study explores this association in Ghana, using epidemiological data at the district level (2015–2018) and data on health, hygiene, and education. We applied a multilevel model and time series decomposition to understand the epidemiological pattern of malaria in Ghana. Then we classified the districts of Ghana into rural, peri-urban, and urban areas using administratively defined urbanization, total built areas, and built intensity. We converted the prevalence time series into cross-sectional data for each district by extracting features from the data. To predict the determinant most impacting according to the degree of urbanization, we used a cluster-specific random forest. We find that prevalence is impacted by seasonality, but the trend of the seasonal signature is not noticeable in urban and peri-urban areas. While urban districts have a slightly lower prevalence, there are still pockets with higher rates within these regions. These areas of high prevalence are linked to proximity to water bodies and waterways, but the rise in these same variables is not associated with the increase of prevalence in peri-urban areas. The increase in nightlight reflectance in rural areas is associated with an increased prevalence. We conclude that urbanization is not the main factor driving the decline in malaria. However, the data indicate that understanding and managing malaria prevalence in urbanization will necessitate a focus on these contextual factors. Finally, we design an interactive tool, ’malDecision’ that allows data-supported decision-making.

## Introduction

Concurrent changes in vector ecology and human dynamics are transforming the risk of malaria prevalence, and urbanization is a key factor in this process [1]. These changes are notable in the global south, where the unprecedented scale and rate of changes in human settlement patterns play an important role in malaria epidemiology [2, 3]. Malaria, especially tropical malaria caused by *Plasmodium falciparum* is commonly regarded as a disease more prevalent in rural areas [4, 5]. The primary carriers of *P*. *falciparum* in West Africa are anopheline mosquitoes with varying frequency and density across seasons and areas. *Anopheles gambiae s*.*l*., for example, is more common in urban Ghana, while *An*. *coluzzii*, *An*. *funestus* and *An*. *arabiensis* are better suited to rural settings [6, 7]. However, increased migration from rural to urban can lead to more malaria cases also in urban settings [3, 8]. In this article, we study the heterogeneity of malaria prevalence over four years in Ghana (urban and rural areas) and examine the association between malaria prevalence and urbanization.

More than 50% of the population of Ghana lives in urban areas and an increase in the proportion of the urban population is likely to continue in the future [9]. By 2050, Ghana’s urban population will be more than double that of its rural population [10]. Like many developing countries, urbanization in Ghana is accompanied by challenges such as traffic congestion, unregulated informal economic activities, and social inequalities. Furthermore, expanding cities tend to maintain and increase rural-urban links and intensify human mobility, as happens in Accra and Kumasi, the two largest urban areas of Ghana [11]. The imminent transition, along with other associated sociodemographic changes and disease ecology, can change the existing burden of malaria [4]. However, little is known about the confluence of urbanization dynamics and the burden of malaria disease in the country.

Urbanization has altered the epidemiology of malaria in Africa cities, yet the findings remain inclusive, mainly due to the varying spatial granularity employed to elucidate the connection between the epidemic and urbanization. Several studies using global to regional methods have argued in favor of a reduction of malaria prevalence in urban areas due to the improvement, accessibility, and availability of health systems [12–14], but others using the country-to-town scale showed that urban dwellers are at increased risk of malaria due to unplanned urbanization, poverty, and lack of sanitation infrastructure [2, 15–18]. These interdependencies between urbanization and malaria prevalence suggest a rather complex and nonlinear association, which we can characterize as a context-dependent association. Therefore, we hypothesize that the most prominent determinants that inflate malaria prevalence vary with the degree of urbanization.

This study aims at identifying the factors that underlie the association between malaria prevalence and degree of urbanization. To this end, we examine the role of urbanization using estimates from census and satellite data and perform a novel spatially detailed examination of urbanization and malaria prevalence on the national scale in Ghana, including the number of cases classified by sex and age at the district level.

## Data and methods

In this section, we briefly describe the dataset used and the statistical analysis performed to show the association between malaria and urbanization. Specifically, we used two spatial and temporal scales to emphasize the context of the association between malaria and urbanization. At the district level (first spatial scale), we analyze the temporal trend that highlights differences between age, sex, and location using longitudinal epidemiological data (first temporal scale). These analyses aim to illustrate the inherent heterogeneity in malaria prevalence across space, time, and within and between socio-demographic groups. More specifically, this heterogeneity serves as a foundation to demonstrate the variability in association between degree of urbanization and disease prevalence. Then, we categorized districts of Ghana (second spatial scale) into urban, peri-urban, and rural using quantitative metrics such as administratively defined urbanization, total built areas, and built intensity. At the typographical level (rural, peri-urban vs. urban), we analyze the association between key extracted features from malaria prevalence time series and factors influencing prevalence utilizing complementary cross-sectional datasets (second temporal scale). These analyses were conducted to explain the variation in factors associated with the increasing prevalence, depending on the degree of urbanization. Finally, we case-studied the Greater Accra Metropolitan and Ashanti regions to show the underlying context of this association. All the statistical analysis were performed using R [19].

### Ethical approval

This study was approved by both the ethics committees of ZEF and the Institute of Statistical, Social, and Economic Research (ISSER), University of Ghana, on 28 September 2018 and 04 February 2020, respectively. Formal consent was not required for this study since this study is a non-human subject study.

### Description of datasets

#### Clinical data

We obtained clinical data from the Ghana District Health Information Management System. The clinical data of malaria caused by *P*. *falciparum* comprised explicit data on the number of confirmed uncomplicated malaria cases by rapid diagnostic tests and microscopy. Data contain anonymized outpatient records and are aggregated by month (12 months for each year), district (n = 216), age categories (n = 11), and sex (n = 2) over four years from 2015 to 2018. Clinical cases were used to calculate the prevalence of malaria.

#### Census and satellite data

We obtained current district boundary data from the Government of Ghana (GoG) website [20] and merged them with clinical data, creating a spatialized version of the clinical data set. We also acquired the 2010 Ghana census, from the Ghana Statistical Service for the previous 170 districts, whose boundary data were gathered from the GoG website [21]. The detailed pre-processing is given in the supplemental text (S1 Text).

In addition to census-based urbanization measurement, we computed two additional urbanization metrics (built intensity and built areas) using a satellite-derived Global Human Satellite Layer dataset (v 1.0) for 2014, obtained from [22].

We estimate malaria prevalence and the density of sociodemographic (sex and age) groups in each district from 2015 to 2018 with a gridded population estimated at 100 m spatial resolution WorldPop dataset [23]. Men and women literacy rates, immunization rates, the proportion of insecticide-treated bed-net possession, the proportion of households lacking toilet facilities, and the proportion of households using an improved water source were aggregated at the district level from the Demographic and Health Survey gridded dataset [24]. Average precipitation from 2015 to 2018 at the district level was estimated from the Global Precipitation Measurement (GPM)-based merged satellite-gauge precipitation estimates in Google Earth Engine. Similarly, we used Landsat 8-derived normalized difference vegetation index (NDVI) to estimate median vegetation cover from 2015 to 2018 at the district level. We complemented the data set with additional gridded data sets such as slope, distance from a body of water, distance from a stream of water, elevation of areas, and reflectance of night light in 2018–19, and distribution of healthcare facilities [25–27].

#### Statistical analysis

*Malaria prevalence in space*, *time*, *and sociodemographic groups*. We developed a three-level model (multi-level model) to examine the heterogeneity in malaria prevalence with respect to sociodemographic groups (age and sex), and location (districts). Detailed identification of temporal and spatial heterogeneity (seasonal signature of the epidemic) was done using seasonal and trend decomposition using locally estimated scatterplot smoothing (STL) global and local Moran I statistics, respectively (S1 Text). The chosen parameters for the STL decomposition are such that the trend-cycle window is determined based on the periodicity of the time series, and a periodic seasonal window is used for seasonal extraction. The specific values of the trend-cycle window are determined automatically based on the data.

The local Moran’s I was calculated to assess the number of spatial clusters in malaria prevalence and establish the local indicators of spatial association (LISA) map to visualize the most significant local spatial autocorrelations. Specifically, Moran’s I metrics assessed the similarity in malaria prevalence between neighboring districts and that was visualized on the LISA map (S1 Fig).

*Operationalization of urbanization in the context of Ghana’s prevalence*. We run a k-means on the three urbanization proxies namely the administratively defined urbanization, the total built areas, and the built intensity to group the Ghana districts into three urbanization areas (urban, peri-urban, and rural) (S2 Fig). The accuracy of the classification was checked with human operators to reduce the risk of misclassifications.

*Contextual Association between Malaria Prevalence and Urbanization*. We used principal component analysis (PCA) to streamline the latent space of malaria prevalence. This involved extracting key features from the time series of prevalence focusing on the first principal component. As key features of interest, we extracted the mean, the maximum, the minimum, and the standard deviation per district. This extraction allows obtaining cross-sectional data that gather 62.30% of the information contained in the longitudinal data set. As the heterogeneity in malaria is peculiar to geographic space [16] and social factors [2], we define the contextual association as the set of conditions beneath the relationship between urbanization and the burden of malaria. To circumvent the collinearity problem that could undermine the accuracy of the machine learning method, we select the variables with a variable inflation factor (VIF) less than 10, widely considered as the threshold indicative of multicollinearity (S3 Fig). After fitting the cluster-specific random forest regression model (RF) for urban, peri-urban, and rural areas, the models were evaluated using a training-testing split of 70% and 30%, respectively (S1 Text). The general performance of the model was examined using the leave-one-cluster-out-cross-validation, where 10-fold cross-validation was performed to determine model performance and reduce model bias.

To better understand malaria in urban areas, we case-studied Accra (Greater Accra) and Kumasi (Ashanti) by map visualization.

To translate our findings into actionable insights and improve preparedness for malaria and other emerging infectious diseases, we created ’malDecision’ (S2 Text), an R-based application. malDecision replicates the analyses undertaken in this study, empowering a larger community of infectious disease researchers, public health officials, and policymakers. malDecision, which is built in R, provides a streamlined way to analysis, visualization, and reporting. Users can run studies and generate configurable reports using a single file that includes predictors, target variables (incidence or prevalence), and the administrative classification of targeted locations. Furthermore, malDecision is regionally adaptable, supporting a variety of geographies and circumstances.

## Results

### Spatiotemporal distribution of malaria epidemics across sociodemographic groups

**Fig 1:**
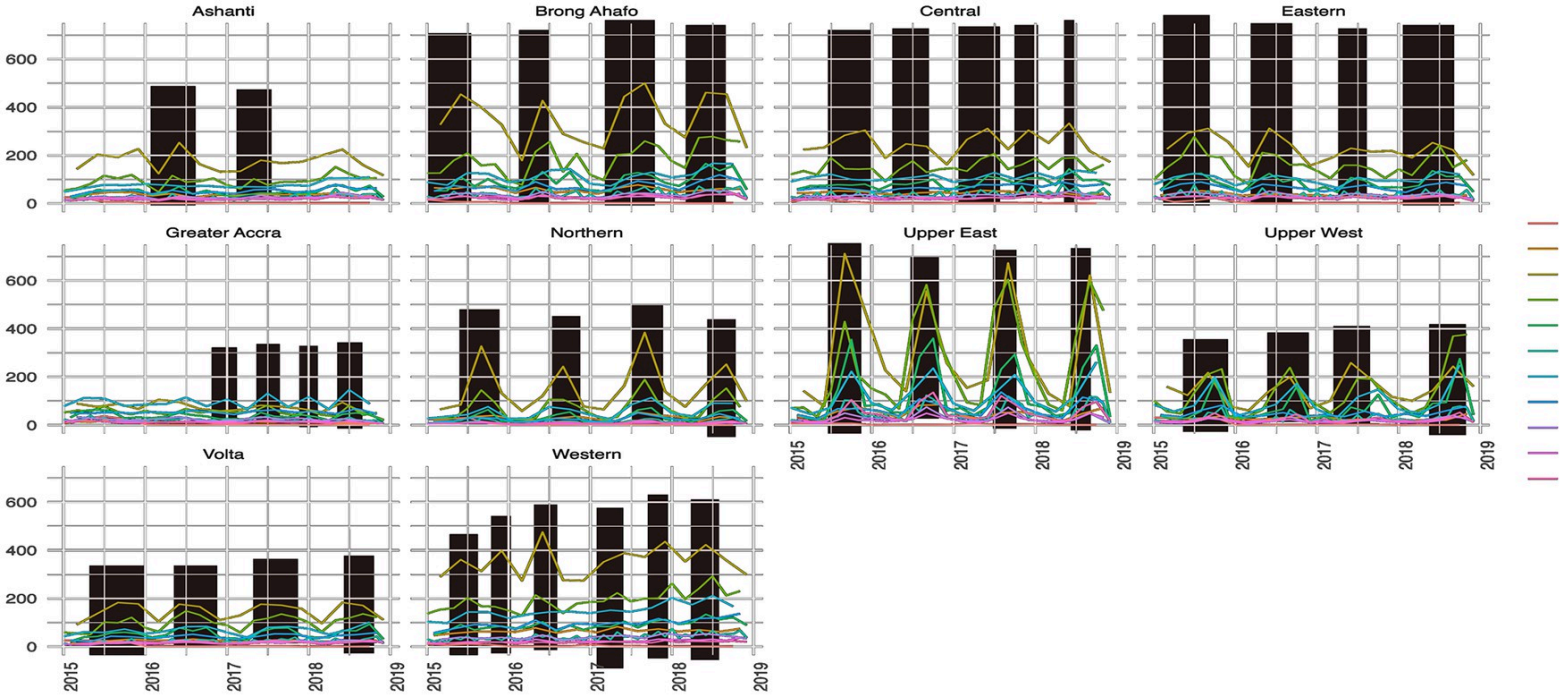
Distribution of the median monthly prevalence in regions by year and age. Squared in pink show the seasonal increase in the prevalence across regions in Ghana.

There is a seasonal signature in malaria prevalence with the highest peak in the rainy season (July to November) (Fig 1). However, this seasonal signature is less perceptible in the districts located in Greater Accra and Ashanti regions. Malaria prevalence varies by age, sex, location, and time. There were >19 million reported clinical cases of malaria between 2015 and 2018. During this period, the highest prevalence was observed among children aged one to five years, and the lowest prevalence occurred in children under a month old across districts of all regions (Fig 1). The prevalence of malaria among women is statistically higher than among men (Table 1). Of all regions, the districts of Upper East region recorded the highest median prevalence of 623 cases per 1000 population for children under five years of age (Fig 1). Across districts and for each year, the median prevalence exceeds 18 cases per 1,000 people (Fig 1). The median prevalence aggregated between districts, age groups, and time points was lowest in the Northern region (18 cases per 1000 population) and highest in districts located in the Brong-Ahafo and Western regions (56 cases per 1000 population) (S1 and S2 Tables). More urban districts located in regions such as Ashanti and Greater Accra had 36 and 26 cases per 1000 population median prevalence, respectively.

**Table 1:** Multilevel model showing the variation over years, sex, and age.

	Null model (Level 1)				Unconditional growth model (Level 2)				Three-level model (Level 3)			
*Predictors*	*Estimates*	*CI*	*p*	*df*	*Estimates*	*CI*	*p*	*df*	*Estimates*	*CI*	*p*	*df*
(Intercept)	71.5	66.18–76.82	**<0.001**	103263	-5732.78	-6725.70 –-4739.86	**<0.001**	103262	-6660.76	-10671.99 –-2649.52	**0.001**	103256
Year					2.88	2.39–3.37Icc	**<0.001**	103262	3.35	1.36–5.34	**0.001**	103256
sex [m]									-2181.44	-6471.70–2108.82	0.319	103256
Age									-138	-233.93 –-42.07	**0.005**	103256
Year × sex [m]									1.08	-1.05–3.21	0.319	103256
Year × Age									0.07	0.02–0.12	**0.005**	103256
sex [m] × Age									231.86	123.29–340.43	**<0.001**	103256
(Year × sex [m]) × Age									-0.12	-0.17 –-0.06	**<0.001**	103256
**Random Effects**												
σ^2^	8171.79				8161.39				9039.23			
τ_00_	1575.54_District_				0.00_District_				0.00_District_			
τ_11_					0.00_District.Year_				0.00_District.Year_			
ρ_01_					0.89_District_				0.00_District_			
ICC	0.16				0.26				0			
N	216_District_				216_District_				216_District_			
Observations	103479				103479				103479			
Marginal R^2^ / Conditional R^2^	0.000 / NA				0.001 / 0.163				0.077 / 0.077			

Across districts, the Global Moran’s I statistic shows significant spatial clustering (Moran’s I = 0.2596, P < 0.01). The local Moran’s I revealed a significant clustering of high prevalence located in 199 districts throughout the country (S4 Fig). This finding suggests that malaria throughout the country is a human reservoir of the plasmodium parasite and consequently argues in favor of possible malaria urbanization (S5 Fig).

Multilevel models showed that there are significant heterogeneities over time, (ICC = 16%) in age, sex, and districts, (ICC = 26%) with 10% variation (model null) variation attributed to differences within districts (Table 1, Level 1, Level 2), respectively.

The average prevalence of malaria within the country estimated by the unconditional means model (level 1) was 71.5 cases per 1000 ± 1.89 (P < 0.0001) (Table 1- Level 1) and the variations between districts explain 16.16% of the total variation (ICC, Table 1). The unconditional growth model (level 2) captured greater variability between districts than the level 1 model (ICC 16.0%, Table 1), and there is a significant variation in malaria prevalence over the years (Table 1). A chi-square test confirms that the three-level additive model offers a better fit than the three-level multiplicative model (P < 0.01). Finally, the level 2 and level 3 models suggest significant interannual heterogeneity, i.e., a seasonal signature of malaria (Table 1). This finding is supported by STL decomposition results, where we observed positive trends in most regions, except for the districts located in Upper West and Upper East regions, as well as seasonal fluctuations (S1 Fig).

### Urbanization in the context of the study

**Fig 2:**
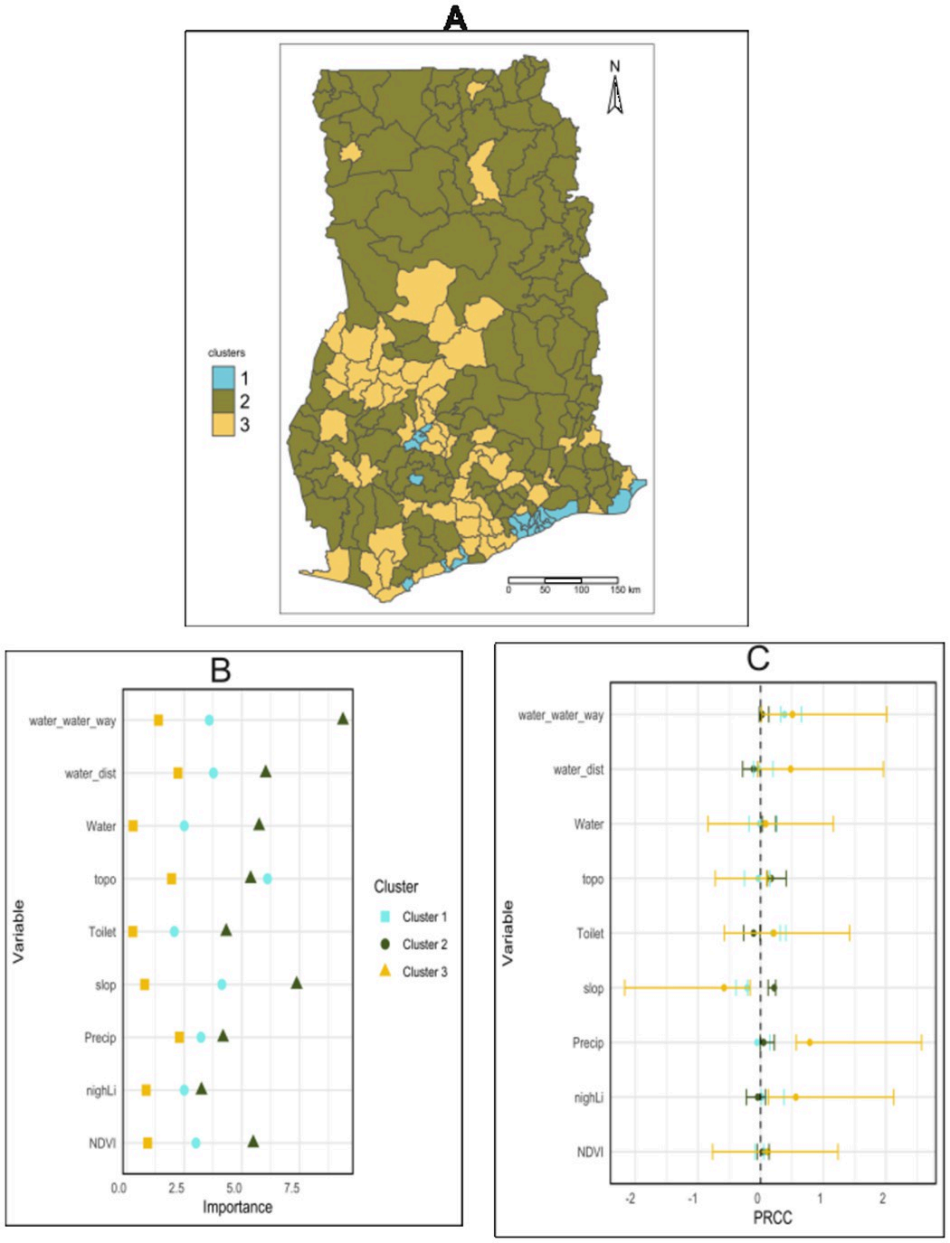
Contextual association between urbanization and prevalence: (A) Clusters of districts by the degree of urbanization; (B) Normalized Variable Importance scores (IncNodePurity) from Random Forest regression analysis of how within each cluster (obtained in [B]), different urbanization features predict average malaria prevalence; (D) Partial Rank Correlation Coefficients (PRCC) of how within each cluster (obtained in [B]), different urbanization features affect median monthly malaria prevalence. The shapefile from Ghana Open Data Initiative [20] (available at https://data.gov.gh/dataset/shapefiles-all-districts-ghana-2012-216-districts).

Twenty-four (24) districts with high built-up areas, intensity, and density are labeled as urban areas (cluster 3). Rural areas are characterized by low-built areas and intensity and low density (cluster 2) whereas moderate proxies are obtained for peri-urban areas (cluster 1) (Table 2, Fig 2A).

**Table 2:** Spatial distribution of the operationalized concept of urbanization post-semi-supervised clustering.

Cluster	Built Areas	Built intensity	Average population density	Number of districts	Denomination
1	24.66	0.05	45.81	64	Peri-urban
2	9.58	0.01	14.02	128	Rural
3	70.85	0.53	57.27	24	Urban

### Contextual association between malaria and urbanization

The extracted features (PCA) showed that the first component is highly correlated with the high value of the extracted features (Supplemental information). Therefore, a district positively associated with the first component will be interpreted as a district with a high prevalence of malaria. We examine the underlying heterogeneities in the extracted feature of malaria prevalence and their association with socioeconomic, environmental, and urbanization determinants (Fig 2A).

In urban areas, the most important determinant explaining the prevalence of malaria is topography, the distance to a body of water, and the distance to a waterway (Fig 2B and 2C). Specifically, closeness to a water body or a waterway is associated with an increased prevalence of malaria, while elevation (topo) is associated with a lower prevalence of malaria prevalence (Fig 2B and 2C).

In peri-urban areas, slope, distance to a water body, distance to a waterway, vegetation cover, and topography influence the prevalence of malaria (Fig 2B and 2C). Specifically, distance to a waterway, elevation (topography), percent change in elevation over a certain distance (slope) and increase in vegetation cover are associated with an increase in malaria prevalence, while distance to a water body is associated with a decrease in malaria prevalence.

In rural areas, slope, distance to a waterway, topography, nightlight reflectance, vegetation cover, precipitation, and distance to a water body impact the prevalence of malaria. Precisely, the distance to a waterway, the increase in night light reflectance, the increase in vegetation cover, and the overall elevation are associated with an increase in malaria prevalence, while the increase in short elevation (slope), the proximity to a water body, and precipitation are associated with low malaria prevalence.

**Fig 3:**
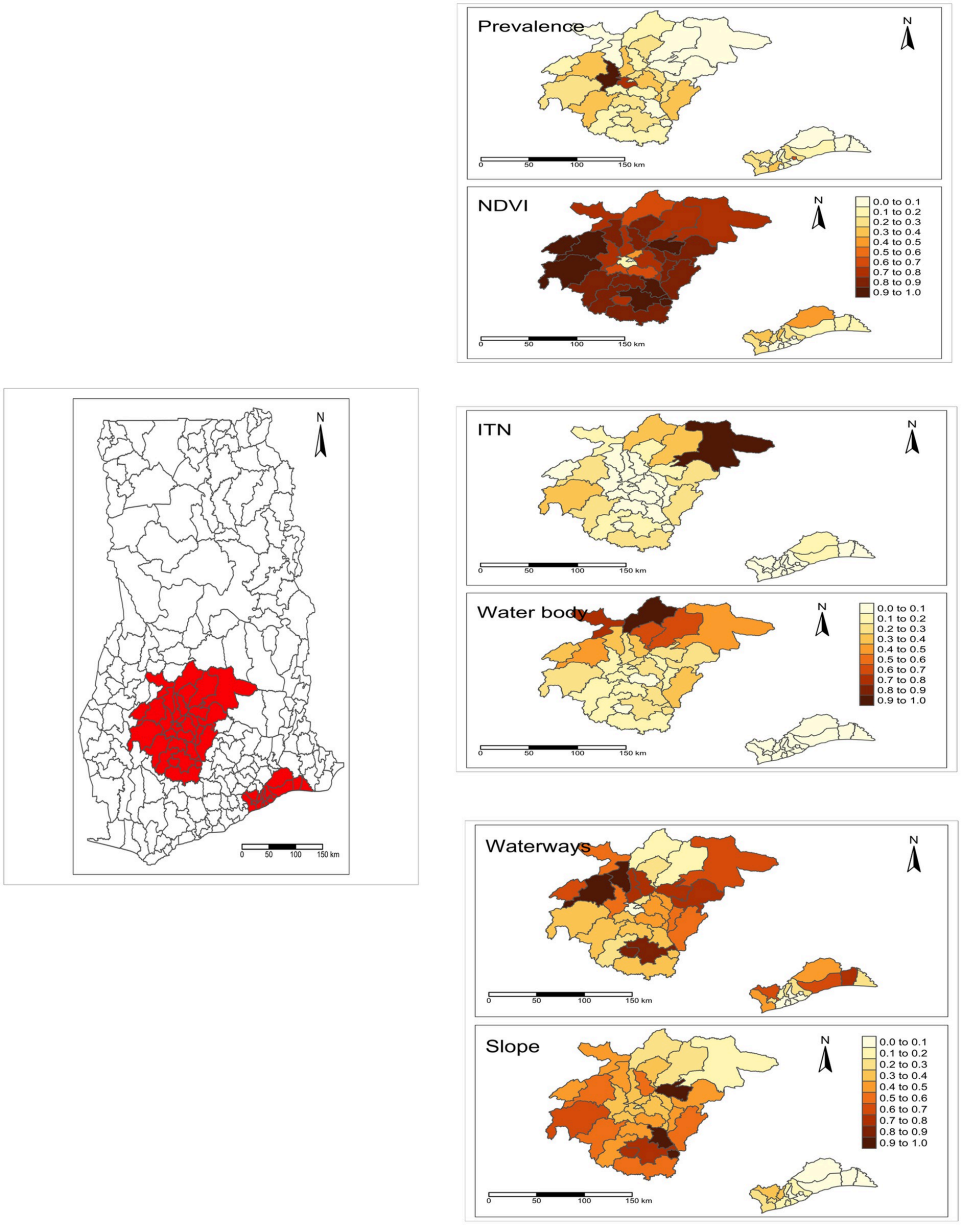
Thematic map of the spatial repartition of the normalized value of key covariate that explains the prevalence of malaria in the districts located in Greater Accra and Ashanti regions where NDVI is Normalized Difference Vegetation Index; ITN, insecticide-treated bed-nets. The shapefile from Ghana Open Data Initiative [20] (available at https://data.gov.gh/dataset/shapefiles-all-districts-ghana-2012-216-districts).

In the districts located in Greater Accra and Ashanti regions, the prevalence is higher in Accra and Kumasi and gradually decreases as one moves away from Accra and Kumasi (Fig 3). Accra and Kumasi also recorded the lowest use of ITN and vegetation cover (Fig 3) in contrast to neighboring districts. Additionally, in Accra and Kumasi, both the proximity to water bodies and the slope are lower compared to neighboring districts.

The cross-validation of the cluster-specific RF revealed that the model for peri-urban areas exhibits higher RMSE (0.90) and MAE (0.71), indicating less accurate predictions. Conversely, the models for rural and urban areas show lower RMSE (0.68 and 0.74, respectively) and MAE (0.53 and 0.69, respectively), suggesting better predictive performance (S3 Table).

## Discussion

### Malaria pattern in Ghana

This study shows that the prevalence of malaria in Ghana has a seasonal signature. However, the seasonal trend observed in rural areas is less prominent in predominantly urban areas such as the Greater Accra and Ashanti regions. Furthermore, we found a heterogeneous distribution in the observed trend within degrees of urbanization where proximity to a body of water and or waterways is associated with a high prevalence of malaria. These results suggest that geophysical factors play a predominant role in malaria prevalence surpassing the alternate between rainfall and dry season, i.e., climatic factors.

The less pronounced signature of seasons in malaria prevalence observed in urban areas can be explained by the constant flow throughout the year of patients from all over the country to reference health centres located in cities [28]. According to the same authors, this inflow curves the epidemic maintaining an equilibrium of prevalence throughout the year in cities.

### Malaria and urbanization in Ghana

Interestingly, the emphasis on the districts located in regions of Ghana revealed some interesting spatial patterns in malaria prevalence, where the centres (Accra and Kumasi) have the highest malaria prevalence compared to districts far away. Identification of significant spatial clusters with Moran’s I metrics, even in districts of traditionally considered urban regions (Accra and Kumasi), challenged the dominant narrative on the intricate relationship between urbanization and malaria, and paved the way for a more nuanced exploration. Furthermore, we found that the increase in malaria prevalence is associated with a lower use of ITN and a lower vegetation cover. This pattern revealed that there is a heterogeneous distribution in both the epidemic and urbanization. Therefore, collapsing the spatial and temporal dimensions that quantify the epidemic and urbanization can lead to spurious conclusions that describe their association. Acknowledging spatial heterogeneity will help to tailor intervention strategies based on the specific characteristics of each region. Regions with different levels of urbanization may require customized approaches to combat malaria effectively. This can be achieved using advanced spatial and temporal analysis techniques that capture the nuances of the relationship.

We also found that the underlying factors that determine the association between malaria and urbanization are not the same. In rural areas, the increase in malaria prevalence is associated with vegetation cover, precipitation, and night light reflectance, suggesting that prevalence is affected by environmental and climatic conditions. In peri urban areas, it is surprising to note that proximity to a water body does not lead to an increase in the prevalence of malaria. This counterintuitive result can underline a man-made environment in which the water body is polluted, preventing malaria vectors from growing under ideal conditions and infecting people. These findings show that malaria in urban settings is more complex than what is implied by considering only urbanization clusters. Therefore, understanding the association between malaria and urbanization requires considering other dimensions such as social and ecological. Examining whether urbanization or urbanicity uniquely increases or decreases malaria prevalence in Ghana requires a contextual lens. In this light, our study highlights the complexity of malaria epidemiology and urbanization and suggests that addressing these complexities requires a multidisciplinary approach.

A major limitation of our study is that the use of historical datasets could preclude the accuracy of our findings. These datasets, especially epidemiological, were mentioned to be not always accurate, since they under-report the number of cases [29]. Efforts were made with the creation of the District Health Information Management System (DHIMS) (source of epidemiological data used in this study) to reduce under-reported cases. However, this data set does not allow one to locate the location of the infection, due to spatial aggregation.

On the other hand, due to the inaccessibility of socioeconomic data at the district level, this study deciphers the intrinsic impact of humans on the prevalence of malaria according to the level of urbanization. Therefore, we develop an application ‘malDecison’ to allow decision-makers to use all their available data to design malaria management based on informed decision-making.

## Conclusion

Our analysis derived a series of findings on malaria prevalence in Ghana, suggesting significant underlying complexities in malaria epidemiology. We found evidence of heterogeneity in the prevalence of malaria that varies in regional and urban contexts. The drivers of malaria prevalence depend on the degree of urbanization, but several confounders operate, opening avenues for future research on the marginal effect of these drivers on the dynamics of malaria disease in urban areas endemic to malaria. Therefore, the observations derived from our analysis would be useful for future modeling to understand the implications of climate change and urbanization that lead to changes in malaria dynamics in Ghana and Sub-Saharan Africa.

## Supporting information

S1 Checklist Inclusivity in global research. (DOCX)

S1 Fig Local Indicator of the spatial association cluster map. The following map (Fig. S1) projects the spatial location of the urban areas in Ghana. Based on the proxy of urbanization used the location of urban areas varied. The shapefile from Ghana Open Data Initiative (available at https://data.gov.gh/dataset/shapefiles-all-districts-ghana-2012-216-districts).
(EPS)

S2 Fig Spatial repartition of urban areas according to the three definitions. This representation illustrates that employing each proxy characterizing urbanization yields distinct results, potentially complicating the quantification of urbanization. There is a need to have a unified definition of the urbanization in the context of this study. The shapefile from Ghana Open Data Initiative (available at https://data.gov.gh/dataset/shapefiles-all-districts-ghana-2012-216-districts).
(EPS)

S3 Fig Plot of the VIF with all the predictors possible of malaria (A) and the after selection of most important predictors (B). We keep the VIF lower than 10 to prevent issues of multicollinearity precluding the performance and accuracy of RF.
(EPS)

S4 Fig Prevalence decomposition showing the seasonality and the trend in each region and the overall country. (EPS)

S5 Fig Spatial distribution of malaria prevalence in Ghana from 2015 to 2018. The shapefile from Ghana Open Data Initiative (available at https://data.gov.gh/dataset/shapefiles-all-districts-ghana-2012-216-districts).
(EPS)

S1 Text Data and methods. (DOCX)

S2 Text malDecision user guideline. (DOCX)

S1 Table Distribution of malaria median prevalence from 2015–2018 per region in Ghana. (XLSX)

S2 Table Cumulative number cases from 2015 to 2018 for each year. (XLSX)

S3 Table Cluster specific random forest regression cross validation. (XLSX)
