# Supplementary material for: Urbanization and malaria have a contextual relationship in endemic areas: A temporal and spatial study in Ghana
Source: PLOS Glob Public Health. 2024 May 30;4(5):e0002871. doi: 10.1371/journal.pgph.0002871 (PMC11139300; doi:10.1371/journal.pgph.0002871)
Supplement: S1 Text — (DOCX) [file pgph.0002871.s007.docx]

**Urbanization and malaria have a contextual relationship in endemic areas: a temporal and spatial study in Ghana**

Merveille Koissi Savi, Bhartendu Pandey, Anshuman Swain, Jeongki Lim, Daniel Callo-Concha, Gbedegnon Roseric Azondekon, Mohammed Wahjib, Christian Borgemeister

**Supplemental materials**

**Text**

**Data**

## ***Census and satellite data***

We obtained current district boundary data from the Ghanaian Government website (GoG) [1] and merged it with clinical data, creating a spatialized version of the clinical dataset. We also acquired the 2010 Ghana census, from the Ghana Statistical Service for the former 170 districts, whose boundary data were gathered from the GoG website [2]. The study addressed the Modifiable Areal Unit Problem (MAUP) by carefully merging and weighting district-level data from different sources, accounting for variations in the number of districts over time and employing spatial join and intersection tools in QGIS to create a spatially explicit and harmonized dataset. To correct for differences in the number of districts, we calculated a weighted average of population counts for each district, where built intensities for 2014 (built area/total area) derived from the Global Human Settlement Layer (GHSL) were used as weights [3]. This approach yielded a combined spatially explicit census and clinical malaria cases dataset. In further analysis, we assumed that the cross-sectional distribution of population counts and urbanization levels did not significantly change since the last population census.

In addition to census-based urbanization measurement, we calculated urbanization metrics using a satellite-derived GHSL dataset (v 1.0) for the year 2014, obtained from [4]. For each district, we calculated the total built area (km^2^) and the built intensity (built area/total area) as two additional measures of urbanization.

**Operationalization of urbanization**

Given that there is a wide spectrum of admitted definitions for urban areas, for the rest of the paper we decided to operationalize our definition of urbanization while combining the built intensity, the built areas, and the density of population. As such, we used a K-means method to classify the district into three categories namely urban, peri-urban, and rural areas. Specifically, urban areas are characterized by a high density of population, a high built intensity, and a high built intensity, whereas in peri-urban all the metrics are moderate, and in rural areas, the metrics chosen are the lowest (Table 2).

**Determinants of malaria**

We estimate the density of sociodemographic groups in each district from 2015 to 2018 with a gridded population estimated at a spatial resolution of 100 m of the WorldPop dataset [5]. Literacy rates for men and women, immunization rates, the proportion of insecticide-treated bed net possession, the proportion of households lacking toilet facilities, and the proportion of households using an improved water source were aggregated at the district level from the gridded dataset of the Demographic and Health Survey [6]. The average precipitation from 2015 to 2018 at the district level was estimated from merged satellite precipitation estimates based on Global Precipitation Measurement (GPM) in Google Earth Engine. Similarly, we use Landsat 8-derived normalized vegetation difference index (NDVI) to estimate the median vegetation cover from 2015 to 2018 at the district level. Furthermore, we estimate the distance to the water body, to a stream of a water body, the average time at night, and the slope [6] as an additional determinant that has previously been used to predict malaria prevalence.

**Missing data and computation of malaria prevalence**

To avoid bias in the interpretation of our findings, we used a chain equation based on a multiple imputation method to estimate the missing observation in the number of clinical cases of malaria [7]. With the complete epidemiological dataset, we computed the prevalence (Eq. 1)

$$Prevalnce=\frac{Number of cases for each month}{Population during the same month} \times{10}^{3}$$

## ***Statistical analysis***

To better understand the spatiotemporal dynamics of malaria prevalence, we used the malaria prevalence dataset from 2015 to 2018 that embeds the location, sex, and age of the recorded cases. We run a multilevel model on this data set to decipher the effect of time, location, and sociodemographic on the dynamics of malaria. To understand the marginal effect of environmental health and hygiene on the features extracted from epidemiological data, we reduce the latent space of the malaria prevalence data set and combine the extracted feature with the cross-sectional determinants. We then proceed with a cluster-specific random forest regression to understand the contribution of each determinant to malaria dynamics in urban, peri-urban, and rural areas.

**Understanding the pattern of malaria prevalence at the country level from longitudinal data**

We fit a single-level model, also denoted the unconditional means model (Eq. 1) to estimate the overall prevalence during data collection for 2015-2018. Here, we evaluated the proportion of variation in the prevalence due to the location using the intraclass correlation coefficient (ICC).

(1)


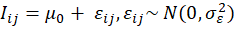


where *i* is the malaria prevalence of district *i* (*i=1, …, 216*) for the year *j* (*j=2015,…, 2018*)*,* is the average prevalence across districts, and is the residual for a specific district and year (difference between the mean prevalence value and the observed value).


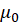

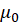

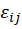

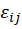


We extended the single-level model to a two-level model, also known as the unconditional growth model (Eq. 2), where the effect of time (random effect) was measured as nested to the district. We computed the ICC of the unconditional growth model to malaria prevalence way across time and location (district).

$$I_{ij}+ \mu_{0}+\left( \mu_{j}+ \epsilon_{ij} \right),$$

$$\mu_{j}\sim N\left( 0, \sigma_{\mu}^{2} \right); \epsilon_{ij}\sim N(0, \sigma_{\epsilon}^{2})$$

(2)

Where represents the average prevalence per year associated with the fixed effect. The variation due to the district is considered as a random effect together with the residual of the model. Both are the random effects of the models assumed to be independent and identically distributed, that is, assumed to follow (~) a normal distribution with mean 0 and variance, respectively and


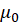

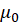

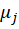

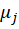

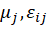

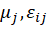

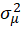

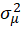

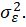

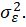


We extended the previous model to a three-level model, adding sex (dummy variable) and age groups (with 11 levels) as fixed effects to compare the effect of sex and age on malaria prevalence. in Eq. 3 becomes


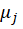

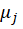


(3)


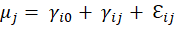

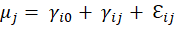


where represents the average prevalence associated with either sex or age; the effects associated with either the sex at the location or the age group at the location. We selected the model that better fit between additive and multiplicative using a chi-square test, where the additive model was the best fit. Similarly, we used the same test to select the better-fit structure of the matrix of variance-covariance for both the residual and the random components of the model (Eq. 3). Then, the matrix of variances-covariance of residuals selected was the first order-autoregressive indicating that the correlation between two observations gets weaker as the distance between them increases, whereas, the unstructured matrix fitted better for the random effects, indicating that there is no constraint across random effects. The statistical difference between the sociodemographic groups and the location was visualized using histograms and maps. To examine the statistical difference between years, we decomposed malaria prevalence time series using an additive decomposition into seasonality, trend, and remainder components, also denoted seasonal trend decomposition using LOESS (STL) [8].


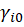

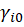

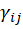

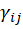


To check consistency in the spatial structure of malaria prevalence, we calculated the Global Moran I statistic using the Queen’s contiguity matrix (Eq. 4).

(4)


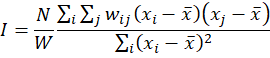


where$N$is the number of districts, $x$ the prevalence of malaria, their mean, $w_{ij}$ the Queen’s contiguity matrix (where $w_{ij}= 0$), and $W$ *is* the sum of all $w_{ij}$.


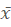

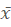


**Contextual Association between Malaria Prevalence and Urbanization**

We extracted key features such as means, maximum, minimum, and standard deviation from the prevalence data set for each district using a first component of the principal component analysis (PCA). This computation allows the transformation of the longitudinal data into a cross-sectional dataset while extracting more than 60% of the information contained in the longitudinal data set. The outcome was used for a cluster-specific random forest (RF), that is, RF regression of health and hygiene and education (the proportion of literate men and women, the rate of immunization, the proportion of insecticide-treated bed-net possession, the proportion of households that lack toilet facilities, the proportion of households using an improved water source and duration of night light duration), environmental (rainfall, vegetation coverage, distance to a water body, distance to a water stream, slope, elevation) and urbanization measures against median monthly malaria prevalence. To avoid the collinearity issue, which frequently affects the accuracy of machine learning algorithms, we move to a subset of the predictors listed above with a variable inflation factor less than 10. Thus, predictors for the RF included the proportion of households without toilet facilities, the proportion of households using an improved water source, the duration of night light, rainfall, vegetation coverage, distance to a water body, distance to a water stream, slope, and elevation (Fig.S5). Each RF regression was done using 10,000 trees to identify which parameters are the strongest predictors of malaria prevalence. We optimized the number of hyperparameters available for splitting at each tree node in the RF using out-of-box error (OBE). The model was evaluated using leave-one-cluster-out cross-validation to determine the RF's overall performance. We estimated model performance using 10-fold cross validation. We calculated Root Mean Squared Error (RMSE), R-squared (R²), and Mean Absolute Error (MAE) during cross-validation.

**References**

1. National Information Technology Agency. Shapefiles of all Districts in Ghana 2010 (216 districts) - Shapefiles of all Districts in Ghana 2012 (216 districts) | Ghana Open Data Initiative. In: Ghana Open Data Initiative [Internet]. 2019 [cited 11 Jan 2023]. Available: https://data.gov.gh/dataset/shapefiles-all-districts-ghana-2012-216-districts/resource/c89cf6a0-ef51-40fc-860d

2. National Information Technology Agency. Shapefiles of all Districts in Ghana (170 districts) | Ghana Open Data Initiative. In: Ghana Open Data Initiative [Internet]. 10 May 2019 [cited 11 Jan 2023]. Available: https://data.gov.gh/dataset/shapefiles-all-districts-ghana-170-districts

3. Pesaresi M, Huadong G, Blaes X, Ehrlich D, Ferri S, Gueguen L, et al. A global human settlement layer from optical HR/VHR RS data: Concept and first results. IEEE J Sel Top Appl Earth Obs Remote Sens. 2013;6: 2102–2131. doi:10.1109/JSTARS.2013.2271445

4. European Union. Index of /ftp/jrc-opendata/GHSL/GHS_BUILT_LDSMT_GLOBE_R2015B. 2019 [cited 11 Jan 2023]. Available: http://cidportal.jrc.ec.europa.eu/ftp/jrc-opendata/GHSL/GHS_BUILT_LDSMT_GLOBE_R2015B/

5. GeoData Institute U of Southampton. WorldPop population database. . 20 Aug 2020 [cited 11 Nov 2020]. Available: https://www.worldpop.org/geodata

6. WorldPop. Global 100m Covariates. University of Southampton; 2018. doi:10.5258/SOTON/WP00644

7. Jakobsen JC, Gluud C, Wetterslev J, Winkel P. When and how should multiple imputation be used for handling missing data in randomised clinical trials – a practical guide with flowcharts. BMC Med Res Methodol. 2017;17: 162. doi:10.1186/s12874-017-0442-1

8. Cleveland RB, Cleveland WS, Terpenning I. STL: A Seasonal-Trend Decomposition Procedure Based on Loess - ProQuest. J Off Stat. 1990;6: 3–73.

9. Government of Ghana. Shapefiles of all Districts in Ghana 2012 (216 districts) | Ghana Open Data Initiative. In: Ghana Open Data Initiative [Internet]. 2012 [cited 22 Dec 2023]. Available: https://data.gov.gh/dataset/shapefiles-all-districts-ghana-2012-216-districts
