# Supplementary material for: Urbanization and malaria have a contextual relationship in endemic areas: A temporal and spatial study in Ghana
Source: PLOS Glob Public Health. 2024 May 30;4(5):e0002871. doi: 10.1371/journal.pgph.0002871 (PMC11139300; doi:10.1371/journal.pgph.0002871)
Supplement: S2 Text — (DOCX) [file pgph.0002871.s008.docx]

**malDecision user guide**

Table of Contents

[***What is malDecision? 2***](#_Toc164001122)

[***Key Features: 2***](#_Toc164001123)

[Analysis: 2](#_Toc164001124)

[Visualization: 3](#_Toc164001125)

[Reporting: 3](#_Toc164001126)

[Example 3](#_Toc164001127)

[***Who Should Use malDecision: 7***](#_Toc164001128)

[Researchers: 7](#_Toc164001129)

[Public Health Officials: 8](#_Toc164001130)

[Policymakers: 8](#_Toc164001131)

[***Get Involved: 8***](#_Toc164001132)

**What is malDecision?**

malDecision is an R-based application developed to translate research findings into actionable insights and enhance preparedness for malaria and other emerging infectious diseases. Our tool replicates the analyses undertaken in our study, empowering a broader community of infectious disease researchers, public health officials, and policymakers.


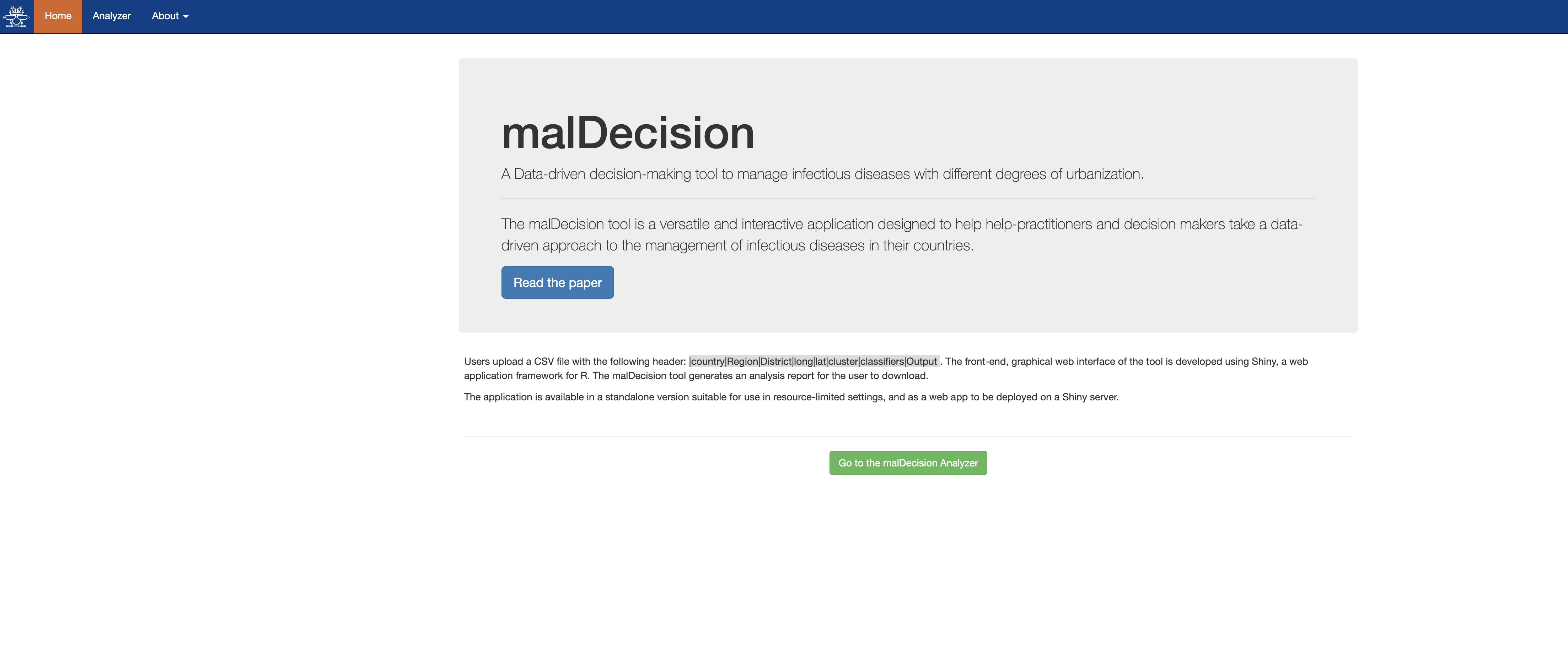


**Key Features:**

**Analysis:**

Input your data and specify your analysis parameters using the provided scripts. Please insert a CSV file formatted as follows:

|country|Region|District|long|lat|cluster| classifiers| Output

Clusters represent the degree of urbanization of the area and classifiers represent the factors that determine access. The output variable represents the variable to be predicted, such as prevalence or incidence

**Visualization:**

Visualize your results using built-in plotting functions or customize your visualizations as needed.

**Reporting:**

Generate comprehensive reports summarizing your findings, which can be easily shared with stakeholders or incorporated into research publications.

**Example**

1. Load the 'example.csv' file
2. Choose a variable cluster that represents the degree of urbanization
3. Select the classifiers (topo, precipitation, etc.) that represent the determinants
4. Choose the output variable (PC1)
5. With a single click, you can run the analysis
6. A cluster-specific Random Forest or a customized report can be withdrawn as an Excel sheet showing the variable of importance. In the custom pdf report containing the descriptive statistics of the dataset, the degree of urbanization is represented spatially. And the variable of importance for each group.

After choosing the country, the study areas were selected. In the left panel, the user can upload his dataset and select his variables.


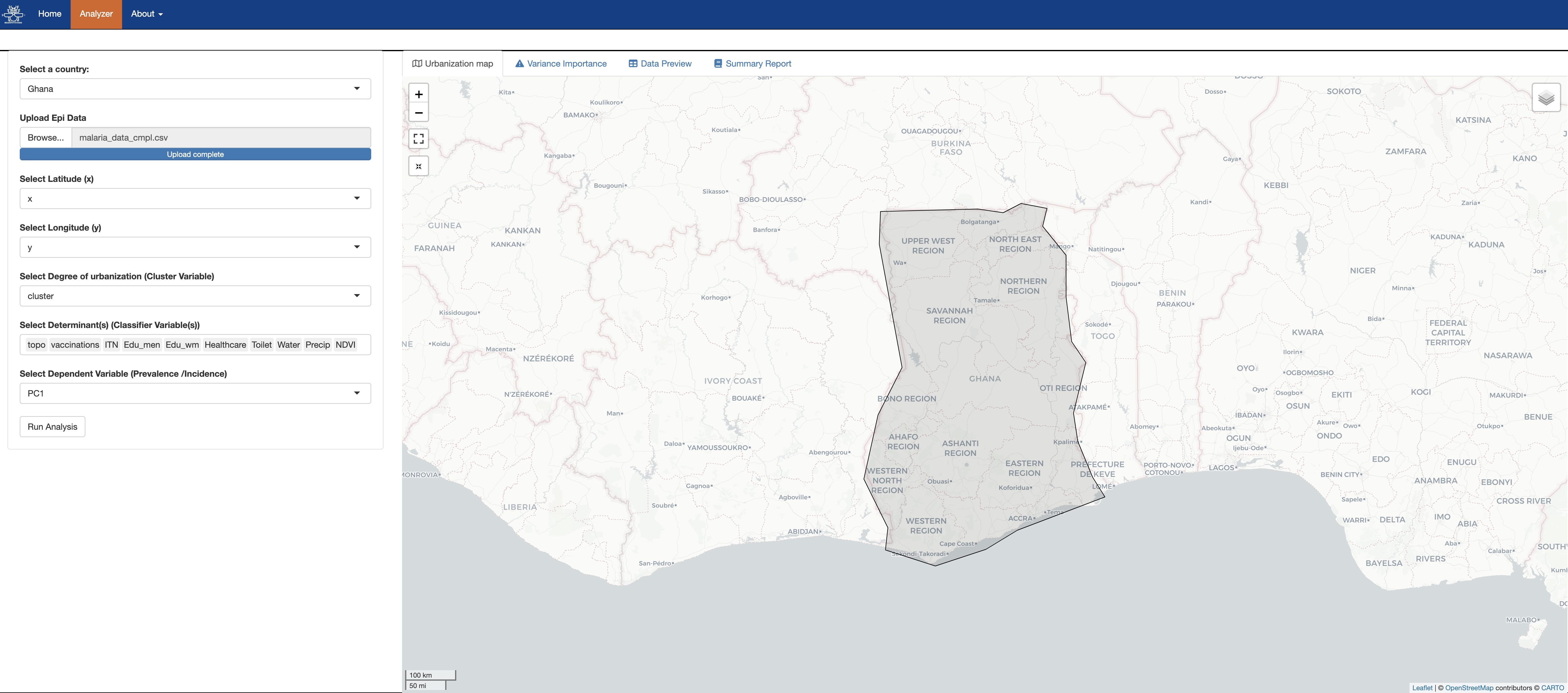


After selecting the variables of interest for the users and clicking on the button run analysis, results are shown starting from the interactive map to the list of variables ordered by importance for each degree of urbanization.

1. Load the file.
2. Choose a variable cluster that represents the degree of urbanization.
3. Select the classifiers (topo, precipitation, etc.) that represent the determinants.
4. Choose the output variable (PC1)
5. With a single click, you can run the analysis.


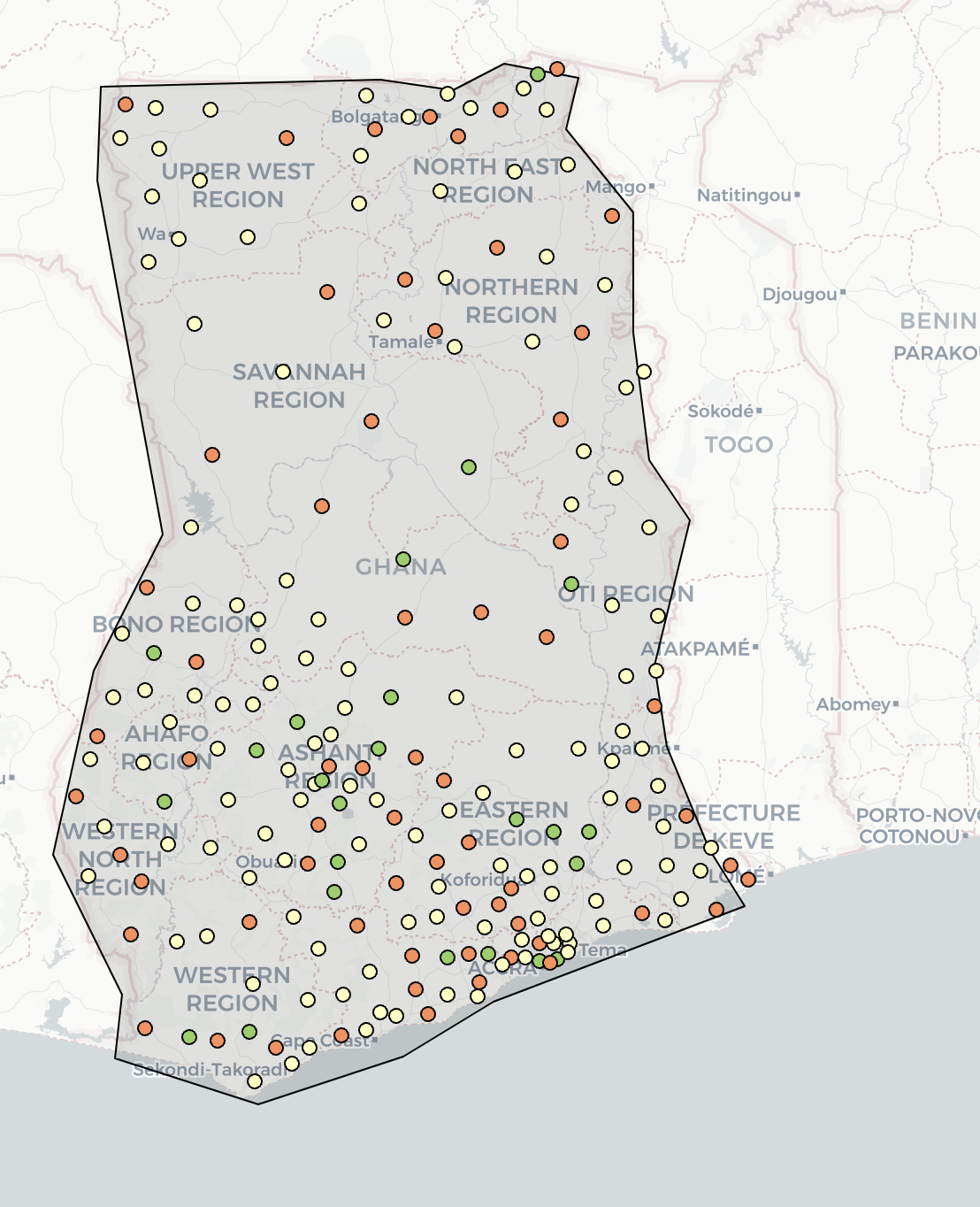


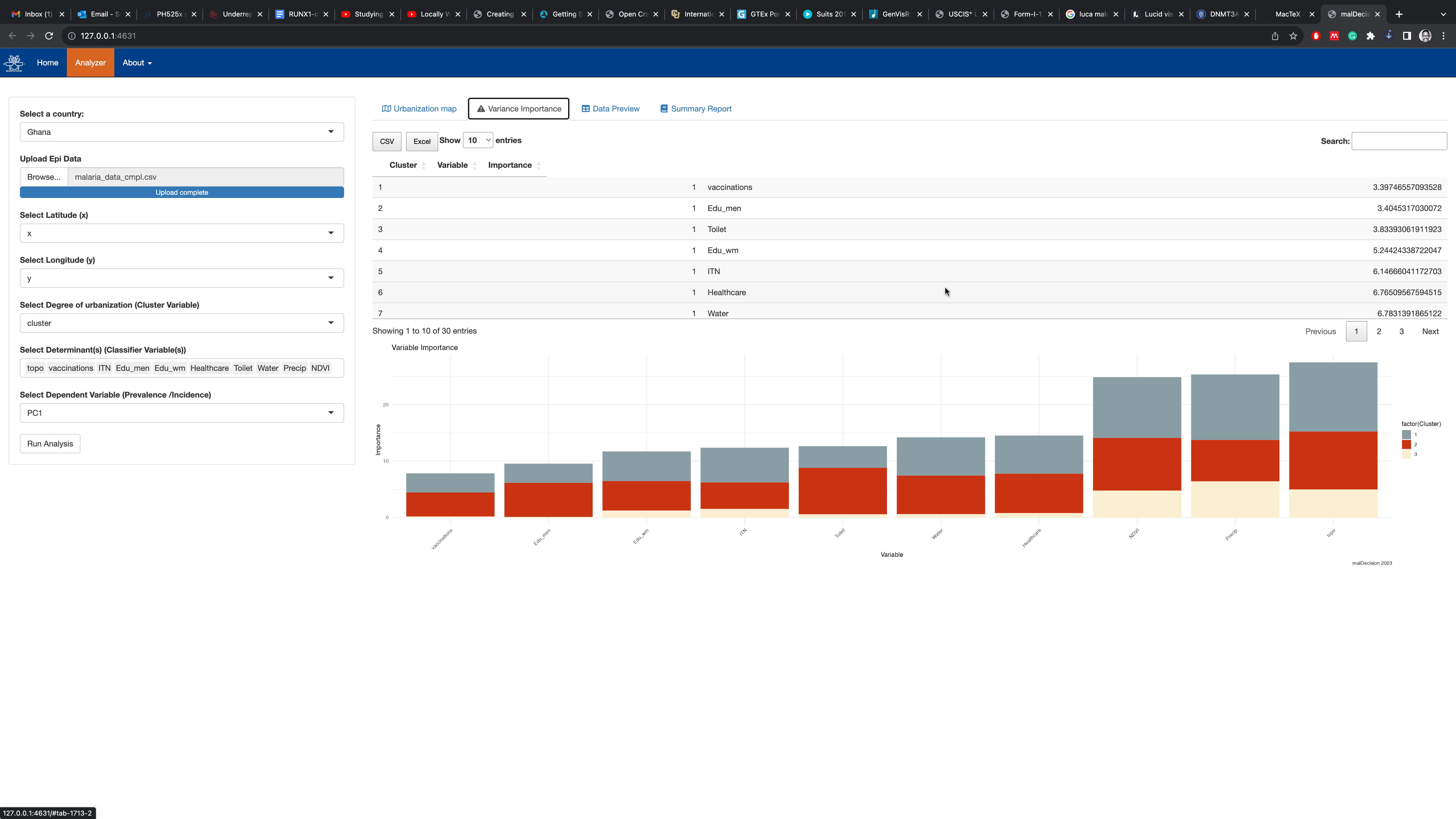


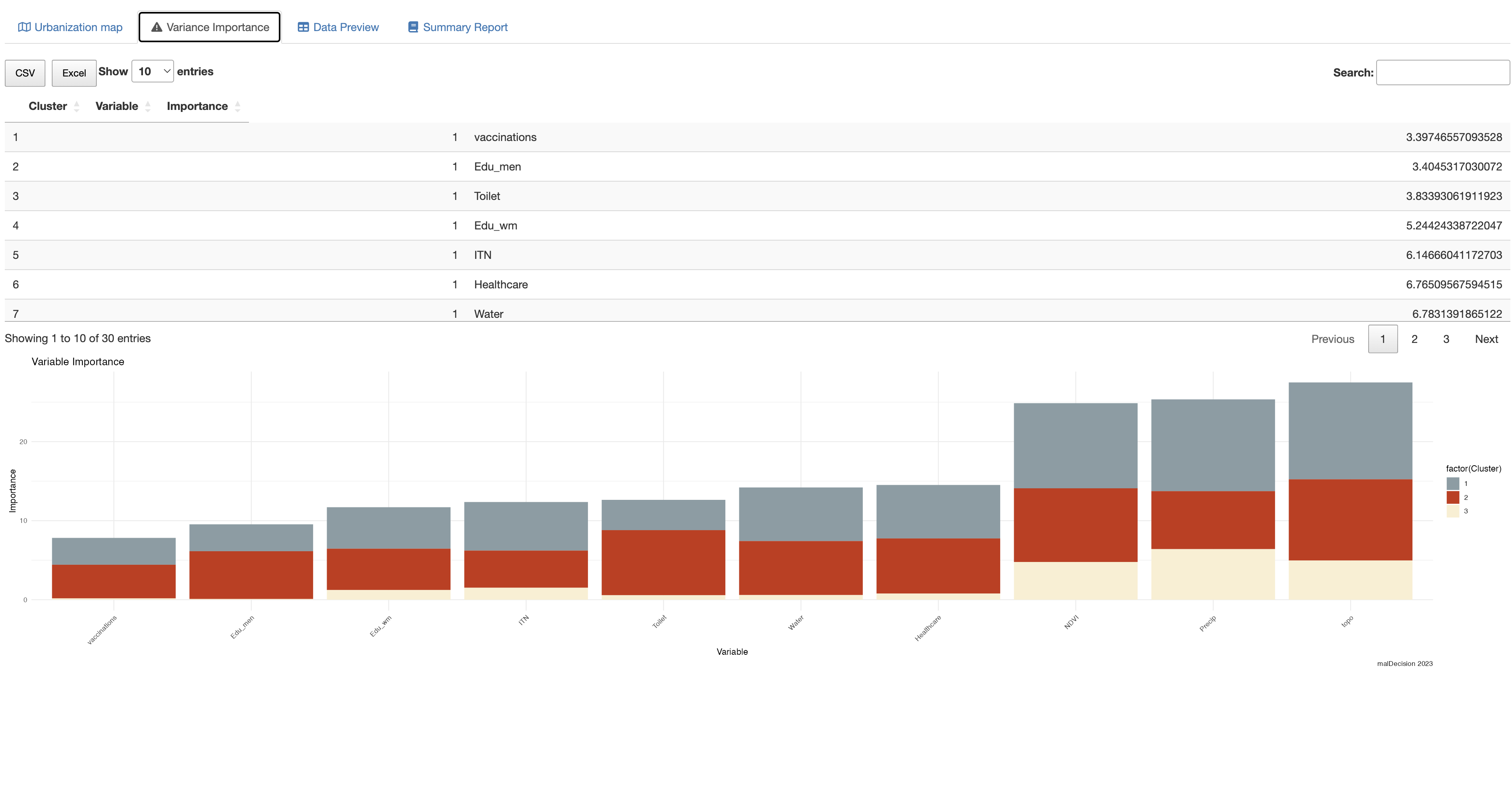


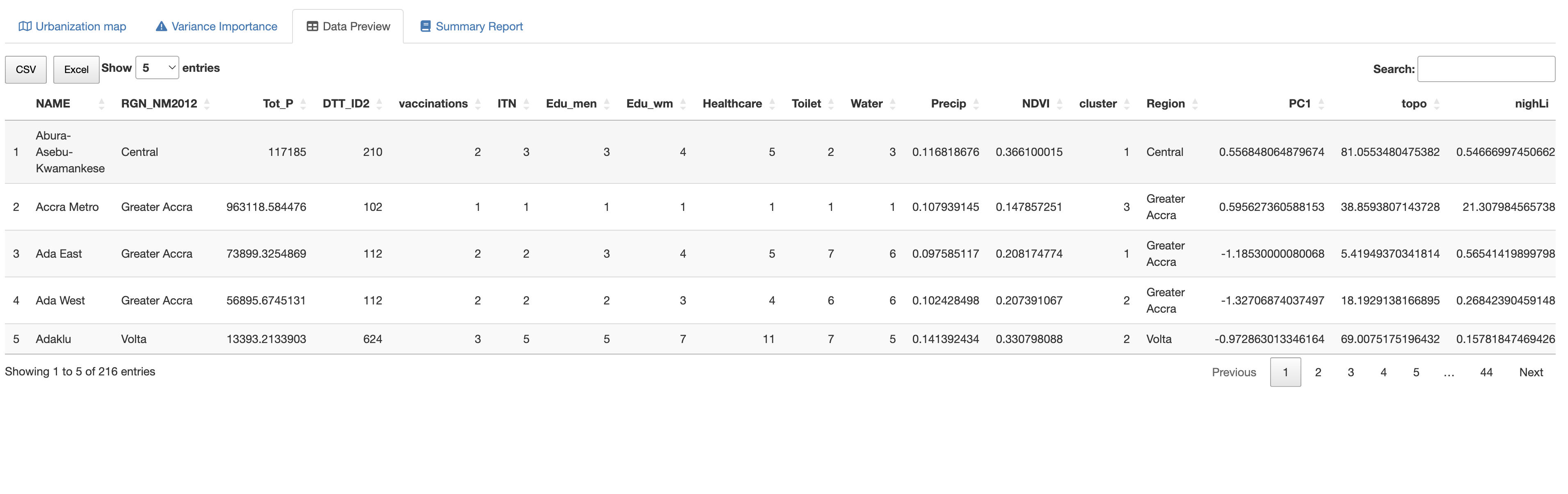


Moreover, users can download a custom PDF report showing the results of the analysis. Besides, users can download the Excel sheet showing the importance of the variables by the degree of urbanization.


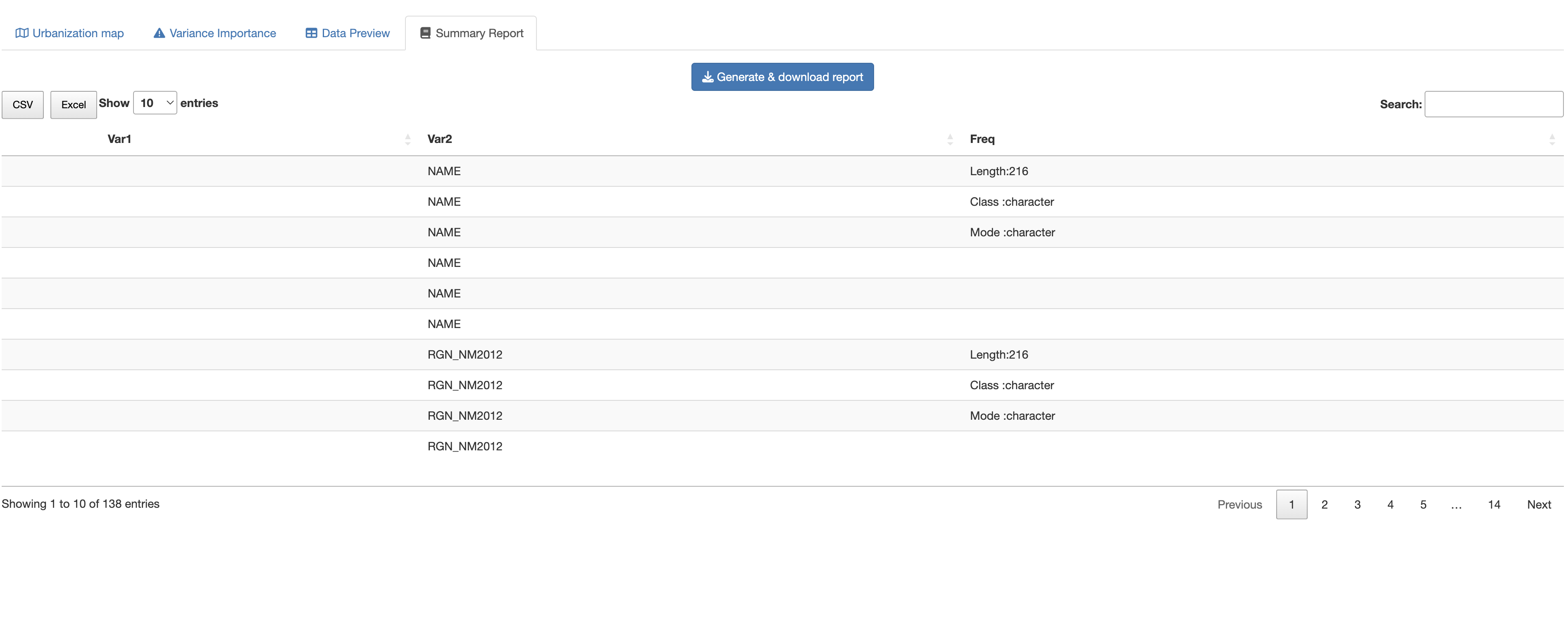


**Who Should Use malDecision:**

**Researchers:**

Conduct sophisticated analyses and visualize results to deepen understanding of infectious disease dynamics.

**Public Health Officials:**

Inform decision-making and resource allocation by analyzing trends and identifying high-risk areas.

**Policymakers:**

Develop evidence-based policies and interventions to mitigate the spread of malaria and other infectious diseases.

**Get Involved:**

We welcome contributions from the community to further enhance the functionality and usability of malDecision. Whether you're interested in adding new features, improving documentation, or reporting bugs, we encourage you to get involved and help us make malDecision even better!
